# Influence of Adipose Tissue on Early Metabolic Programming: Conditioning Factors and Early Screening

**DOI:** 10.3390/diagnostics13091510

**Published:** 2023-04-22

**Authors:** Maria Puche-Juarez, Juan M. Toledano, Julio J. Ochoa, Javier Diaz-Castro, Jorge Moreno-Fernandez

**Affiliations:** 1 Department of Physiology, Faculty of Pharmacy, Campus Universitario de Cartuja, University of Granada, E-18071 Granada, Spainjjoh@ugr.es (J.J.O.); javierdc@ugr.es (J.D.-C.); 2 Institute of Nutrition and Food Technology “José Mataix Verdú”, University of Granada, E-18071 Granada, Spain; 3 Nutrition and Food Sciences Ph.D. Program, University of Granada, E-18071 Granada, Spain; 4 Instituto de Investigación Biosanitaria (IBS), E-18016 Granada, Spain

**Keywords:** fetal programming, adipose tissue, pregnancy, adipogenesis, obesity

## Abstract

Background: Obesity and being overweight have become one of the world’s most severe health issues, not only because of the pathology but also because of the development of related comorbidities. Even when children reach adulthood, the mother’s environment during pregnancy has been found to have a significant impact on obesity prevention in children. Thus, both maternal dietary habits and other factors such as gestational diabetes mellitus, excessive weight gain during pregnancy, smoking, or endocrine factors, among others, could influence newborn growth, adiposity, and body composition at birth, in childhood and adolescence, hence programming health in adulthood. Methods: The aim of this review is to analyze the most recent human studies on the programming of fetal adipose tissue to determine which modifiable factors may influence adiposity and thus prevent specific disorders later in life by means of a bibliographic review of articles related to the subject over the last ten years. Conclusions: The importance of a healthy diet and lifestyle not only during pregnancy and the first months of life but also throughout childhood, especially during the first two years of life as this is a period of great plasticity, where the foundations for optimal health in later life will be laid, preventing the emergence of noncommunicable diseases including obesity, diabetes mellitus type 2, hypertension, being overweight, and any other pathology linked to metabolic syndrome, which is so prevalent today, through health programs beginning at a young age.

## 1. Introduction

Adipose tissue is a complex organ that plays a critical role in the regulation of energy and glucose homeostasis throughout the body, storing energy in the form of lipids and producing a slew of bioactive factors that regulate a variety of metabolic pathways. It begins to form inside the mother’s uterus during the months of pregnancy, and precursors of fat locules can be found around the 14th week of gestation [1], although the second trimester of gestation is the most crucial moment for its development [2]. There are many circumstances that will affect the amount of it, and several studies have found that the adiposity during prenatal life can condition it across the life and thus the appearance of various disorders at birth, in childhood, and in adulthood [3].

### 1.1. Fetal Programming

The fetal origin hypothesis proposes that the nutritional and hormonal environment in utero causes changes in gene expression that may compromise the fetus’ health [4], leading to adaptive physiological changes known as epigenetic alterations occurring when DNA and the proteins that interact with it undergo chemical modifications without changing the genetic coding [5].

Fetal programming is a process of early-life adaptation to environmental conditions, such as nutrition, that can alter gene expression and affect the functions and structures of various organs and tissues, increasing the susceptibility and likelihood of developing certain metabolic disorders in adulthood [6].

Epidemiological studies on neonatal and adult mortality have led to the development of the Developmental Origins of Health and Disease (DOHaD) theory proposed by Baker et al. [7], which describes how fetal nutrition at various stages of pregnancy can be linked to various birth phenotypes related to adaptations associated with changes in placental and fetal endocrine function and metabolic abnormalities. This fetal programming may influence neonatal growth and development as well as the prevention of chronic non-communicable diseases such as obesity, overweight, diabetes mellitus type 2, hypertension, and other common conditions nowadays.

Obesity and being overweight are described by the World Health Organization as an abnormal or excessive accumulation of fat that may be harmful to health [8]. Individual patterns of body fat distribution vary greatly based on heredity, hormone sensitivity, sex, and race [9], and not all fat is created equal, with some fat deposits having metabolic benefits [10].

### 1.2. Adipose Tissue

The different types of adipose tissue are composed of adipocytes, interstitial fibroblast cells, and progenitor cells, producing an energy storage organ in the form of triglycerides [11]. This is the only tissue that can modify its volume after achieving adult size [9] and is well equipped to react to hormonal and sympathetic stimuli on a biochemical and molecular level [12].

**Figure 1:**
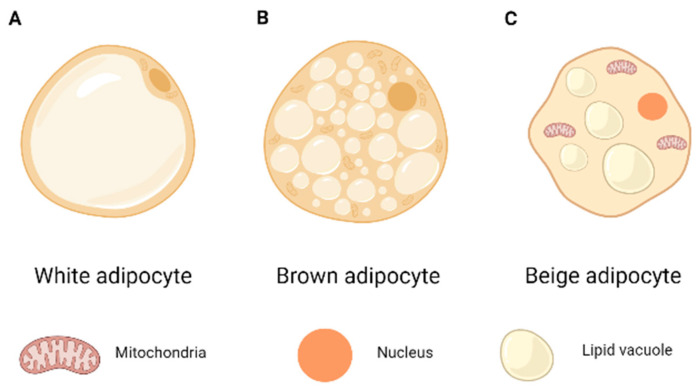
Types of different adipocytes: (**A**) white adipocyte; (**B**) brown adipocyte; (**C**) beige adipocyte.

White adipose tissue (WAT) is mainly composed of adipocytes containing a large unilocular lipid droplet (Figure 1A). It is common to categorize WAT locations as visceral (in the trunk cavity) or subcutaneous (below the skin). As there is no standard method for defining the anatomical location of each WAT depot, depot designations frequently change between studies [10]. The gluteal-femoral depot, located around the hips and thighs in humans, is the most common subcutaneous depot. Numerous epidemiological studies have linked visceral fat to a higher risk of metabolic disorders, as well as subcutaneous white adipose tissue to better insulin sensitivity and reduced the risk of type 2 diabetes mellitus [13]. Its main function is energy storage, but it also has an endocrine function by secreting hormones and cytokines, which regulate metabolism and nutrition [14].

Brown adipose tissue (BAT) is made up of brown adipocytes that are rich in mitochondria, which explains their brown coloration [15], and contain numerous lipid droplets (multilocular). It is also specialized in energy expenditure [10], unlike the preceding one (Figure 1B). It is found in the axillary, cervical, perirenal, periadrenal, and interscapular regions and is involved in thermogenesis [16]. Recent research has discovered it in human adults following exposure to cold, nursing, and exercise [15]. Brown adipocytes, unlike white adipocytes, generate heat through a process known as active thermogenesis [17]. The function of these adipocytes is closely related to Uncoupling Protein 1 (UCP-1), which is a mitochondrial protein expressed only in brown adipocytes and, therefore, is considered a mitochondrial marker of BAT [15]. UCP-1 is located on the inner mitochondrial side, and it works by uncoupling oxidative phosphorylation, regulating thermogenesis, producing heat, and dissipating the electrochemical proton gradient over the mitochondrial membrane but not generating ATP [18].

The third type of adipose tissue has recently been discovered, known as “beige”, which is a stage halfway between WAT and BAT and begins to express more UCP-1 than WAT. When compared to conventional brown adipocytes, adipocytes at this stage show an overlapping but unique gene expression pattern. Both express a core program of thermogenic and mitochondrial genes [19]. These adipocytes, while similar to BAT adipocytes in terms of form and the presence of many lipid vacuoles, are located in the subcutaneous portions of white adipose tissue in a different anatomical position (Figure 1C). Adipocytes in this tissue display a similar set of molecular markers as white adipocytes, but during transdifferentiation, they take on a brown adipocyte-like expression pattern, which is thermogenic and reflects increased energy expenditure and oxygen intake [20].

In humans, adipose tissue appears between 14 and 24 weeks of gestation, and by the beginning of the third trimester, adipose tissue deposits are fully established [21]. In mice, the finest development of adipose tissue coincides with the periods of greatest plasticity during pregnancy and lactation, when hormonal, nutritional, and epigenetic signals influenced by the mother are likely to program permanent alterations in the adipose tissue of the offspring [22].

### 1.3. Endocrine Function

Adipose tissue was formerly assumed to be a source of triglyceride-based energy stores, with the only known function being the body’s use of these energy stores via lipogenesis or lipolysis. In the mid-1990s, a protein factor produced by adipose tissue with action on the central nervous system was identified; it was called leptin [23]. From this point on, a series of factors secreted by this tissue, known as adipokines, were identified, and it was classified as an endocrine tissue.

Adipokines are a set of self-, para-, and endocrine-acting peptides, hormones, and molecules involved in signaling the functional state of adipose tissue to target cells in the brain, liver, pancreas, blood vessels, muscles, and other tissues [24]. They are involved in the regulation of several physiological processes in adipose tissue and at the systemic level. Their secretion modulates appetite, metabolism, innate immune function, and reproduction, and their abnormal secretion contributes to numerous obesity-associated diseases [13].

The programming functions of some of the most significant adipokines are briefly described in Table 1.

### 1.4. Role of the Placenta in Adipose Tissue

This is a highly multifunctional organ. It controls gas exchange, protects the fetus from the immune system of the mother, and transports carbon dioxide and excretions from the fetus to the mother. Furthermore, it acts as a nutrient sensor, regulating the transfer of nutrients from the mother to the fetus and adjusting nutrient transfer capacity to match the fetus’s growth requirements. Moreover, it is a transitory endocrine organ, secreting various hormones and cytokines that can affect maternal and fetal metabolism [37].

**Figure 2:**
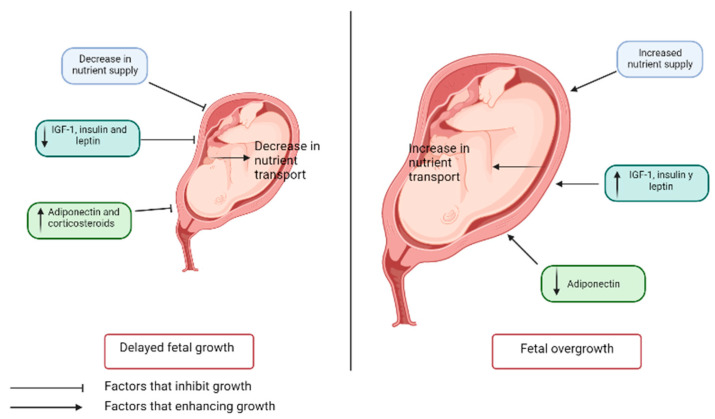
Factors involved in fetal growth retardation and fetal overgrowth.

As a result, when the supply of nutrients and the ability to oxygenate the placenta are compromised, placental functions such as nutrient transport and placental growth can be inhibited, resulting in reduced fetal growth. Maternal concentrations of IGF-1, leptin, and insulin are also related to fetal growth, being lower in fetal growth restriction and, conversely, higher in fetal overgrowth, contrary to what happens with adiponectin, which is increased in IUGR and decreased in overgrowth [38]. These adipokines, as explained before, modulate processes such as appetite, and their plasma concentrations are related to the development of diseases such as obesity. On the other hand, under conditions of malnutrition, sensitivity to nutrients may become exaggerated, contributing to fetal overgrowth [39] (Figure 2).

As obesity and overweight are reaching pandemic levels worldwide, especially in industrialized countries, and not only these diseases but also those related to them and in general the so-called metabolic syndrome, which also includes hypertension, diabetes mellitus type II, and heart disease. Moreover, these diseases are appearing earlier and earlier in life, such as in childhood, and the most recent research shows that any event that happens during pregnancy will program the health of the future baby, especially in non-communicable diseases. Adipose tissue, as an energy storage organ as well as an endocrine organ, has a relevant role in the appearance of these diseases, and the way it is formed and programmed will play a part in the appearance of these diseases. Thus, this review aims to discuss the influence of adipose tissue on early metabolic programming, reviewing the main factors that may condition this process and how they could modulate the offspring’s future health and disease, specifically focusing on hormonal, nutritional, epigenetic, and external factors, and also including breastfeeding as a critical moment in fetal programming. A better understanding of how this tissue is shaped and modulated may be beneficial in curbing the rise of obesity and increasingly prevalent metabolic diseases, which can be prevented during pregnancy. Major findings of early metabolic programming studies focused on adipose tissue are shown in Table 2.

## 2. Materials and Methods

The search dated from September 2022 to January 2023. Website search engines and electronic databases such as PubMed, Scielo, and Cochrane were used, including consistent key words such as early programming, metabolic programming, adipose tissue, adiposity, and fetal growth. Dates of all publications were noted if available. The inclusion criteria were manuscripts with expertise in the field of nutrition. Exclusion criteria were manuscripts written in a language other than English or Spanish, webinars, blogs, or podcasts; and manuscripts written before 2012. The keywords included were: “Fetal programming”; “Adipose tissue”; “Adiposity”; “Obesity”; “Breastfeeding”; and “Pregnancy”. After the screening of titles and abstracts, 48 manuscripts were selected for further examination. Subsequently, 3 manuscripts were excluded due to a lack of relevance; therefore, 45 papers were included in this narrative review. Text from each manuscript was extracted into an Excel document and reviewed to find repeated issues by the authors. The topics, main objectives, and keywords were agreed upon by all authors.

## 3. Results and Discussion

### 3.1. Endocrine Factors Regulating Adipose Tissue Programming

Currently, the global spread of obesity has a pandemic dimension. Numerous studies have focused on the possible effects of maternal obesity on the risk of disease in future generations. Obesity in pregnancy and gestational diabetes mellitus (GDM) are linked to an increase in birth weight, excessive feeding of the newborn, and rapid growth in the first few months of life, all of which predispose the neonate to fat accumulation. In this regard, some studies have found a link between maternal endocrine function and birth weight.

An important element associated with birth weight as a function of gestational age is the insulin-like growth factors IGF-1 and 2, which are expressed in the placenta. Syncytiotrophoblast secretes placental growth hormone (GH) into the maternal circulation during pregnancy. Placental GH stimulates maternal secretion of insulin growth factor 1 (IGF-1), which promotes nutrients transfer across the placenta and fetal growth. From mid- to late-pregnancy. IGF-1 and 2 levels increase from mid- to late-pregnancy, although not both correlate with fetal growth. IGF-1 levels in mid- to late-pregnancy indicate increased placental and fetal growth, reflected in birth weight, whereas IGF-2 levels appear not to be associated with these [40], although low levels of IGF-2 do appear to be associated with reduced lean mass [41]. Other body composition measurements, including body fat percentage and total fat mass, are positively linked with IGF-1, indicating that IGF-1 may have a predominant role in adipose tissue growth [42]. Most of the studies analyzed focus on birth weight or body composition, finding clear evidence of the association between IGF-1 and fetal growth [40,42]. However, a study conducted during the first two months of life in which body composition was assessed in relation to umbilical cord IGF-1 and IGF-2 levels at birth found that levels of both were not a determinant of body composition at two months after birth [41].

In 2015, Putet et al. [43] hypothesized that protein intake could produce an effect on growth velocity in infancy mediated by IGF-1. In the study, they fed infants for the first 4 months of life exclusively on normal-protein formula (2.7 g protein/100 kcal) or low-protein formula (1.8 g protein/100 kcal). Body composition parameters were measured at 4, 6, 9, 12, and 36 months of age, and they found that increased protein did not affect IGF-1 concentrations, although infant length and head circumference were affected, suggesting that other factors than IGF-1 play an important role in growth velocity.

Another hormone involved in fetal growth and development is maternal leptin, a polypeptide hormone secreted by adipocytes and expressed in adipose tissue that promotes energy expenditure and inhibits feeding intake under physiological conditions; however, it is increased in pathological situations such as obesity. The levels of this hormone in the different trimesters of pregnancy and in the umbilical cord at birth are related to different parameters of body composition. Early pregnancy maternal leptin levels are linked to newborn abdominal circumferences and newborn subscapular skinfold thicknesses, and late pregnancy maternal leptin levels are linked to triceps skinfolds, whereas fetal leptin levels are linked to birth weight, specific markers of regional adiposity, and markers of central and overall adiposity [44,45].

During pregnancy, excessive weight gain can lead to many complications for both mother and fetus. In 2016, Solis-Paredes et al. demonstrated in a study of healthy mothers that excessive maternal weight gain during pregnancy is associated with greater levels of leptin in the mother and higher levels of LDL in the fetus. Moreover, mother resistin demonstrated an inverse relationship with fetal LDL, indicating that maternal obesity may actively influence fetal programming by altering the fetal lipid profile [46].

Another biomarker that can be affected by excessive weight gain during pregnancy is insulin. Blood glucose and insulin resistance have different effects during pregnancy, with maternal insulin resistance being an independent predictor of neonatal adiposity in the first half of pregnancy; later, maternal blood glucose, even within the normal range, facilitates fat accumulation [47].

Adiponectin, the endocrine connection between maternal adipose tissue and fetal development, is a significant component of the route for fetal overgrowth. During pregnancy, a lower adiponectin concentration is associated with gestational diabetes; both parameters are associated with increased fetal growth; conversely, an increase in adiponectin in overweight and obese women during pregnancy is associated with a smaller fetal abdominal circumference in the last weeks of gestation [48].

Recent studies suggest that, in addition to glucose, fetal growth is related to maternal triglycerides [49], but these must be hydrolyzed by placental lipoprotein lipase (pLPL). pLPL hydrolyzes maternal triglycerides, both dietary chylomicrons and low-density lipoproteins, to free fatty acids, which promotes fetal fat accumulation by increasing the free fatty acid pool for placental absorption [50].

### 3.2. Nutritional Factors and the Adipose Tissue Programming

It is well established that both overnutrition and undernutrition lead to endocrine changes associated with adiposity in adulthood. Many studies aim to understand how diet-induced nutrients and hormones increase or decrease the risk of obesity during childhood and adulthood.

A classic Mediterranean diet, for instance, is characterized by a high consumption of olive oil, fruits, vegetables, legumes, nuts, and whole-grain items. Dietary patterns also have a significant effect on fetal enlargement throughout pregnancy. Consuming fish in moderation and limiting the intake of red and processed meat has been associated for many years with the prevention of obesity in adults, and thanks to Chatzi et al. (2017) [51] and their study on two cohorts from different regions and countries (Massachusetts and Crete), we know the relationship between adherence to a traditional Mediterranean diet during pregnancy and lower adiposity not only at birth, but also in childhood, where body composition parameters were remeasured in children aged 4-7 years. In addition, improving dietary pattern and lifestyle, including physical activity during pregnancy, in overweight or obese Australian mothers is associated with less thigh fat and less subscapular adiposity in the fetus at 36 weeks of pregnancy [52].

In contrast, in another study conducted in Sweden in 2016 by Claesson et al. [53], the focus was on obese mothers that received weekly dietary advice during pregnancy and every 6 months for the first two years of life and participated in aqua aerobics classes and followed up with the babies until the age of 5 years. Overall, there was no difference between the intervention group compared to the control group who simply received standard care during pregnancy with respect to children’s body mass index (BMI) at 5 years of age. These results are supported by other studies that also intervened on groups of overweight or obese mothers and found no consistent associations between maternal diet and fetal adiposity [54].

Not only is it interesting to change lifestyle habits, such as nutrition and sport, in a holistic way, but focusing on specific nutrients can also help improve the body composition of the newborn. In the case of DHA, docohexanoic acid is of vital importance during pregnancy, as it helps the neonate’s nervous and visual systems to develop properly. Furthermore, high levels in obese women or women with gestational diabetes mellitus in the last weeks of pregnancy (around 36 weeks) have been positively associated with lower adiposity in children at 2 and 4 years of age, results obtained when exclusive breastfeeding is also taken into account, suggesting that DHA acts through breastfeeding [55]. Sodium and saturated fat intake are associated with increased adiposity in infants at 6 months of age [56].

High protein intake and high protein/carbohydrate ratios are associated with increased abdominal fat in the fetus in the last weeks of gestation, providing evidence that maternal diet during pregnancy is a modulator of infant body composition [57]. Although similar results were found in a study of obese mothers, adiposity was unrelated to the amount of macronutrients in the diet when adjusting for pregestational BMI [58], suggesting that maternal obesity is a powerful confounder of this relationship.

On the other hand, the nutritional needs of the mother and fetus during pregnancy vary throughout the pregnancy, being different in the three trimesters of gestation and therefore affecting the composition of the fetus differently during the 9 months. Thus, maternal diet during early pregnancy does not seem to have an impact on the newborn’s weight, but maternal diet in late pregnancy is associated with the baby´s body composition in the first months and years of life, with dietary fats (total fat, polyunsaturated fatty acids, and saturated fatty acids) being a negative factor for adiposity, especially in areas of abdominal subcutaneous fat; protein is linked to BMI at 3 and 5 years of age; and dietary fiber is positively associated with all body composition parameters at 1, 3, and 5 years of age [59].

The glycemic load of the diet is another modifiable factor that has shown great benefits for adiposity and body composition in infants. The ROLO study (Randomized Control Trial of Low Glycemic Index Diet in Pregnancy) is a randomized controlled trial that studies the differences between a low glycemic index (GI) diet and a normal diet in neonates. The main results were that a low GI diet is associated with a smaller thigh circumference in neonates [60], but there is no association between this diet and adiposity at 6 months of age [44]. Total lipids in the mother’s blood at the time of conception and in the umbilical cord were also measured, finding a positive association between these and the anthropometry and birth weight of the baby [61]. Additionally, neonatal central adiposity is positively associated with saturated fats and negatively with circulating polyunsaturated fats in the mother [44], concluding that the body composition of offspring during pregnancy can be modulated by dietary changes. A good strategy would be dietary advice to lower the glycemic load of intakes, as it is a non-harmful diet and can be beneficial for infant adiposity.

Consequently, maternal obesity, being overweight, and gestational diabetes mellitus that may result from these pathologies are strong predisposing factors for increased infant adiposity [62] and fetal overgrowth [63] not only at birth but also in the first years of life. In addition, excessive weight gain during pregnancy predisposes the baby to be born with higher fat mass compared to mothers with controlled weight gain [64], which, coupled with an elevated obese or overweight BMI, suggests rapid fetal growth predisposing to the development of obesity later in life [65]. As for gestational diabetes mellitus, correct treatment of this helps to reduce weight gain during pregnancy, although it is still quite high with respect to healthy pregnant women, which is related to increased infant body mass, the risk of being large for gestational age, and higher birth weight [66]. In contrast, when it comes to type 2 diabetes mellitus, treatment of this and adherence to a healthy diet are related to reduced fetal growth [67].

### 3.3. THE Link between Breastfeeding and the Adipose Tissue

The first few months of life comprise a critical period for the newborn since it is a period of high plasticity during which the newborn’s health is influenced by many external variables, not only during this period but also later in life. Breastfeeding is of great interest and is recommended as the exclusive method of feeding during the first 6 months of life, as in addition to providing the nutrients necessary for proper growth, it also provides substances such as immunoglobulins, oligosaccharides, and growth factors.

Recent studies have tested whether breastfeeding during the first months of life can be considered a public health measure to reduce childhood obesity. The protein concentration of breast milk is slightly lower than that of commercial formulas fed to infants as a substitute for human milk, so it could be deduced that this difference could reduce the development of adipocytes. However, the results are contrary to this hypothesis in children at 12 years of age, whose mothers were advised on exclusive breastfeeding. This did not prevent being overweight or obesity or reduce IGF-1 levels [68]. Even in a prolonged follow-up of these children, no decrease in adiposity was achieved in the intervention group, with this group having a higher prevalence of obesity than the control group [69]. Similar results are found in a 2015 study in Brazilian children, where, in addition to breastfeeding recommendations, advice was also given on the introduction of complementary feeding at 6 months of age to the control group, but no difference was found regarding adiposity between the two groups at 4 years of age [70].

In contrast, positive results are found if different commercial formula milks are compared, but with different protein contents, namely a common commercial one with 2.7 g of protein/100 kcal and a low-protein one with 1.8 g of protein/100 kcal. Infants fed the low-protein formula had lower weight gain between 3 and 6 months, and their protein biomarkers were more similar to those who were exclusively breastfed [71].

### 3.4. Environmental Factors Regulating Adipose Tissue Programming

Pre-, peri-, and postnatal factors are key in determining adiposity and obesity in children and even in adulthood. However, not only are the first 2 years of life important for the development of these metabolic impairments, since factors such as the origin of the parents, their overweight, maternal smoking during pregnancy, and the rapid weight gain of the baby are determining factors for childhood overweight because children exposed to obesogenic environments are at risk of developing obesity [72]. A factor closely related to the adipose tissue of the newborn is the mother’s BMI, which indicates obesity or overweight. Because of this element, the child’s health will be affected not only by the disorder itself but also by the rise in complications from other conditions that it brings with it. A good example of this is the SASR-CoV-2 infection. Obesity itself is a pro-inflammatory state that has been a problem during COVID-19, increasing disease complications in these patients explained by increased inflammation and increased oxidative stress [73]. Furthermore, the prevalence of obesity is higher in women infected with COVID-19 during pregnancy than in those with a normal BMI, so even though it is difficult to predict which symptoms will occur and who will suffer them during infection, obesity and overweight are predisposing factors to complications [74]. Therefore, adequate vaccination during pregnancy, especially in women who are overweight or obese, is key to reducing the likelihood of infection and, in the event of infection, reducing its complications [75].

On the other hand, it has been investigated how complementary feeding after weaning can reduce the risk of obesity, proposing that the so-called “Baby-led weaning”, a technique that consists of the introduction of non-liquid solid foods directed by the baby (eating autonomously with their own hands), may improve energy self-regulation and reduce the risk of obesity. Although studies available have not found an improvement in BMI with this feeding technique, it is associated with reduced food cravings and could therefore be a positive method [76].

### 3.5. Early Screening for Risks Associated with High Adipose Tissue in Offspring

As mentioned above, certain pregnancy-associated diseases are correlated with increased comorbidities at birth, as well as increased adiposity in the newborn and even obesity in later years. Therefore, early detection of the possible risk of this type of gestational disease is crucial for proper perinatal health.

In this regard, GDM, a pathology that develops between the second and third trimesters of gestation, causes endogenous fetal stimulation of insulin and IGF-1 in the placenta, which has been linked to macrosomia [77], as well as an increased risk of hypertension and insulin resistance in childhood and adolescence [78]. Currently, the detection of this pathology is not standardized in all countries and the different obstetric associations, with the most commonly used techniques for detection being different glucose tests and measurement of blood glucose values [79] and reaching a higher BMI, which can be associated with another independent factor, but it has been demonstrated that in children born to the same mother, children born after the mother developed GDM have reached a higher BMI in adolescence than their siblings born after a normal pregnancy [80]. Nowadays, there are many parameters and criteria for the diagnosis of DMG used by more than 30 associations and obstetrics organizations. In addition, these associations also differentiate who should be tested, differentiating between universal, population-wide or risk-based screening, which means that the test is only performed if the pregnant woman meets a number of criteria considered to be at risk such as: previous history of GDM; previous high blood glucose levels; pre-diabetes/impaired glucose metabolism; being older than 35 years; family history of diabetes mellitus; body mass index of 25–30 kg/m^2^, previous macrosomia; signs of insulin resistance such as polycystic ovary syndrome (PCOS) and acanthosis nigricans, medications: corticosteroids, antipsychotics; history of congenital anomalies; pregnancy-induced hypertension; history of stillbirth or miscarriage; multiparity 2; history of preterm birth; history of neonatal death; current smoking; current alcohol use; excessive weight gain in index pregnancy; minority ethnic family background with high prevalence of diabetes (Latino, Native American, Caribbean, Chinese, Asian, Indian subcontinent, Aboriginal, Torres Strait Islander, Pacific Islander, Maori, Middle Eastern, and non-white African); history of PAOD (peripheral arterial occlusive disease); CVD (cerebral vascular disease); hypertension (>140/90 mmHg or on treatment for hypertension); HDL cholesterol level <35 mg/dL (0.90 mmol/L) and/or a triglyceride level > 250 mg/dL (2.82 mmol/L); a sister with hyperglycemia in pregnancy; physical inactivity; short stature; multifetal pregnancy; vitamin D deficiency; maternal history of low birth weight; and HbA1c 5.7% [81]. The screening criteria for the diagnosis of GDM for the most common associations are listed in Table 3. These tests or markers allow the disease to be recognized once it has already occurred, but early prediction will allow better management, early treatment, and even prevention. Some biomarkers have been proposed for this purpose, such as 1,5-anhydroglucitol. This competes with very high levels of glucose for reabsorption in the kidney, so that low levels may reflect hyperglycemia, and its measurement during the first trimester has been validated as a screening method for later GDM [82]. Adipokines such as leptin and adiponectin, mentioned above, are also associated with the development of GDM due to their pro- and anti-inflammatory roles, respectively, and their role in insulin sensitization. Thus, the plasma adiponectin/leptin ratio (<0.33) during the first 6–14 weeks of gestation has also been studied as a strong predictor of subsequent GDM [83]. On the other hand, not only plasma or blood markers are being studied for the detection of GDM. One of the best known risk factors associated with GDM has been BMI, but this index is somewhat unspecific as it does not take into account muscle mass or fat deposits, so other methods are currently being studied, such as ultrasound to measure the depth of visceral adipose tissue, which, when measured early in pregnancy, helps in the early recognition of GDM [84]. Even better results have been found in predicting GDM compared to the aforementioned BMI [85]. Therefore, pregnant women classified as being at high risk for GDM during early pregnancy on the basis of a HAT measurement may benefit from early prevention strategies or healthier disease management [86]. The lack of consensus not only between the organizations but even between different hospitals from the same country leads to the prevalence of these diseases, problems with their management, more complications, and the inefficacy of the treatments.

In addition, a recent meta-analysis reviewing a total of 39 articles concluded that women who develop GDM have a 9-fold increased risk of developing DM2 in the future [87]. Some of the major associations, such as the American Diabetes Association, recommend monitoring these women and their progression with diabetes during the first few weeks postpartum [88]. However, there is little monitoring of these women after childbirth [89]. Breastfeeding has also been postulated as a mechanism for postpartum improvement of gestational diabetes, improving insulin sensitivity [90,91], which could be an extra help together with the mother’s lifestyle habits to prevent the onset of T2DM, as well as the offspring born from a mother who developed GDM and with exclusive breastfeeding had less risk of developing DMT2 later in life [92]. Not only early detection but also adherence to the treatment should be ensured, as correct treatment can determine metabolic programming and the development of adipose tissue in both the mother and the fetus [93].

Another disease occurring in pregnancy that may condition birth and the development of the newborn during infancy, increasing the risk of weight gain and increased adipose tissue deposition, is pre-eclampsia, characterized by the onset of hypertension after 20 weeks of gestation and end-organ dysfunction [94]. Several studies have investigated the relationship between different fat compartments measured by various methods such as tomography or magnetic resonance imaging, ultrasonography [95], and ultrasound [96] to estimate visceral fat and its association with the development of hypertensive disease throughout pregnancy, proving to be a useful tool for early detection.

Therefore, adequate screening for these diseases may benefit maternal health and fetal development during pregnancy, as well as prevent other diseases such as overweight or obesity later in life. Adequate postpartum follow-up is also necessary to make sure that T2DM does not develop or, if it does, to find it as early as possible in order to control it properly.

### 3.6. Limitations

The main limitation of this review is the heterogeneity of the studies reviewed. The studies have been carried out in different populations from all over the world and with various sample sizes. In this regard, nutritional factors are very different between countries, so the different data obtained from the research cannot be extrapolated to the whole population. Additionally, different cultures and lifestyles may cloud the results obtained from breastfeeding studies. On the other hand, as mentioned in the same section, the diagnosis of diseases such as GDM is different depending on which obstetric association or health body is consulted, and there are even differences between associations in the same country, which makes it difficult to reach a conclusion on how to diagnose it or how to treat it.

**Table 2:** Total number of articles reviewed.

Authors and Year	Sample Information	Study Design	Major Findings
Endocrine factors regulating adipose tissue programming			
Luo ZC et al. (2012) [40]	307 women from Canada: 27 GDM 280 no diabetics	Observational	Higher IGF-I levels indicate increased placental and fetal growth. Higher maternal and fetal IGF-I levels may explain fetal hypertrophy in children of mothers with GDM.
Hawkes CP et al. (2019) [41]	601 children born to healthy mothers (Ireland) 317 boys 284 girls	Observational	Increased IGF-1 concentrations are associated with higher fat mass and higher free fat mass at birth. IGF-2 is associated with reduced lean mass.
Kadakia R et al. (2016) [42]	112 pairs of women-children, healthy mothers, from Chicago	Observational	IGF-1 is an important and independent factor affecting neonatal body composition.
Putet G et al. (2016) [43]	238 healthy full-term infants - 74 F1 - 80 F2 - 84 BF	- F1: formula milk, 1.8 g protein/100 kcal - F2: formula milk, 2.7 g protein/100 kcal - BF: breastfeeding, reference group	Factors different than IGF-1 may play a role in determining growth velocity.
Horan MK et al. (2014) [44]	280 mother-children. ROLO clinical trial - 138 cases - 142 control	Low glycemic index dietary advice vs. usual prenatal care	- No effect of a low glycemic index diet during pregnancy on newborn adiposity. - Diet during pregnancy, especially saturated fat and sodium is associated with infant adiposity at 6 months after birth.
Donnelly JM et al. (2015) [45]	- 185 newborns - ROLO clinical trial - 89 cases - 96 control	Low glycemic index diet during pregnancy vs. normal dietary guidelines	Fetal leptin in the umbilical cord significantly associated with birth weight.
Solis-Paredes M et al. (2016) [46]	67 women at term that gave birth by caesarean	Observational	Maternal adiposity status may play an active role in regulating fetal lipid profile and, consequently, fetal programming.
Crume TL et al. (2015) [47]	804 women-children from Colorado	Observational	Insulin resistance in early pregnancy is an independent predictor of neonatal adiposity. Maternal circulating lipid levels have a relatively modest impact on neonatal fat accumulation and adiposity.
O’Brien CM et al. (2019) [48]	911 women in the “Standard care” group of the LIMIT, OW or OB clinical trial.	Standard healthcare recommendation from their hospitals.	Increased adiponectin concentrations are associated with a smaller abdominal circumference in women with OW or OB.
Barbour LA et al. (2018) [49]	54 women from Colorado - 27 normal weight/26 babies - 27 obese/19 babies	Observational	Postprandial TGs are positively related to excess weight in the newborn and therefore their control during pregnancy may prevent excess adiposity in the NB and decrease the risk of obesity.
Heerwagen MJR et al. (2018) [50]	20 women from the Colorado Multiple Institutional Review COMIRB Board - 13 normal weight - 7 obese	Observational	Placental LPL activity correlates positively with adiposity in the newborn.
Nutritional factors and adipose tissue programming			
Chatzi L et al. (2018) [51]	- Project viva: 997 women from Massachusetts - The Rhea cohort: 569 women (Creta)	Observational	Greater adherence to the Mediterranean diet during pregnancy is associated with lower adiposity in offspring.
Grivell RM et al. (2016) [52]	Australian women OW or OB - 935 cases (lifestyle advice) - 912 control (standard care)	- Lifestyle: diet and lifestyle intervention - Standard: usually standard care recommendation from their hospitals.	Lower thigh fat mass and lower subscapular adiposity in children born to obese mothers with diet and lifestyle intervention.
Claesson IM et al. (2016) [53]	302 children-obese mother from Sweden - 137 cases - 165 control	Weekly visits with midwife during pregnancy to change nutrition and fitness habits + aqua aerobics classes.	Dietary advices and practicing physical exercise during pregnancy are not related to the BMI of children at 5 years old.
O’Brien CM et al. (2018) [54]	721 pregnant women in the “Standard Care” group. LIMIT clinical trial.	Usual health recommendation from their hospitals.	No association between maternal diet and fetal adiposity at 28 and 36 weeks’ gestation.
Foster BA et al. (2017) [55]	63 obese women or women with GDM (double-blind clinical trial)	Supplementation with 800 mg/day of DHA.	DHA supplementation during pregnancy is associated with lower adiposity in the offspring.
Horan MK et al. (2016) [56]	280 mother-children. ROLO trial - 138 cases - 142 control	Low glycemic index dietary advice vs. usual prenatal care	Diet during pregnancy, especially saturated fat and sodium is associated with infant adiposity at 6 months after birth.
Blumfield ML et al. (2014) [57]	179 Australian pregnant women from “Women and Their Children’s Healthy Study”	Observational	The body composition of the fetus may be modulated by the mother’s nutritional intervention during pregnancy.
Crume TL et al. (2016) [58]	1410 healthy pregnant women from Colorado	Observational	Regardless of pregestational BMI, increased intake of macronutrients except protein is associated with an increase in neonatal fat mass.
Brei C et al. (2018) [59]	208 healthy pregnant women	- 1.2 g long-chain n-3 PUFAs at 15 weeks and 4 months postpartum and dietary advice for a low arachidonic acid diet. - Control group: normal recommendations.	- Maternal diet in early pregnancy has no significant influence on infant birth weight. - Protein was found to be negatively associated with BMI at 3 and 5 years.
Donnelly JM et al. (2015) [60]	- 185 newborns - ROLO clinical trial - 89 cases - 96 control	Low glycemic index diet during pregnancy vs. normal dietary guidelines	Low GI diet is associated with a smaller thigh circumference in neonates, but not with adiposity at 6 months of age.
Geraghty AA et al. (2016) [61]	331 mothers-children ROLO clinical trial	Low glycemic index diet during pregnancy vs. normal dietary guidelines.	Blood lipid concentrations at the end of pregnancy and in the cord are associated with offspring anthropometry. Maternal TG concentrations are associated with birth weight.
Berglund SK et al. (2016) [62]	PREOBE clinical trial (cohorts) - 1: Normal weight 132 - 2: Overweight 56 - 3: Obese 64 - 4: GDM 79	Observational	Children born to obese or overweight mothers have higher birth weights than those born to normal weight mothers.
Poprzeczny AJ et al. (2018) [63]	912 women in the Standard Care group of the LIMIT study (102 developed GDM)	Guidelines and usual recommendations of their hospitals for pregnancy.	Increased maternal BMI in overweight/obese women is associated with increased fetal growth, but not with increased fetal adiposity.
Nehab SR et al. (2020) [64]	124 women-children from Rio de Janeiro	Observational	Women with excessive weight gain have children with higher body mass and higher fat mass, compared to mothers with adequate or insufficient weight gain in pregnancy.
Savage JS et al. (2019) [65]	26 women from USA with OW or OB - 13 cases - 13 control	Dietary advice through a weekly interview with a nutritionist, and physical activity advice.	Individualized feeding in women with OW or OB shows preventive effects on fetal growth velocity and thus lower risk of OW or OB of the baby.
Blackwell SC et al. (2016) [66]	841 women from a clinical trial of women with GDM. Texas - 431 cases - 410 control	Dietary advice and treatment for GDM.	Excessive weight gain is associated with an increase in the baby’s body mass, risk of being large for gestational age, and higher total birth weight.
Ásbjörnsdóttir B et al. (2019) [67]	219 women with DM2 in Copenhagen - 97 intervention - 92 control	- Motivational interviewing towards adherence to a healthy diet - Diabetes care routine in both groups	There is a trend towards a lower prevalence of fetal overgrowth when encouraged to improve adherence to a healthy diet in addition to routine diabetes care, in women with DM2 and the children born to them.
Link between breastfeeding and the adipose tissue			
Martin RM et al. (2013) [68]	17,046 newborns from PROBIT clinical trial recruited between 1996 and 1997. (Belarus) - 7405 intervention group - 6474 control group	- The intervention group received advice on exclusive breastfeeding. - The control group received the usual advice from their health center.	An intervention to improve the duration and duration of breastfeeding did not prevent overweight or obesity, nor did it affect IGF-I levels, among these children at 11.5 years.
M Martin, et al. (2017) [69]	17,046 newborns from PROBIT clinical trial recruited between 1996–97. (Belarus) - 7405 intervention group - 6474 control group	- The intervention group received advice on exclusive breastfeeding. - The control group received the usual advice from their health center.	Increasing the duration and exclusivity of breastfeeding did not decrease adiposity in children at 16 years of age or blood pressure.
Schwartz R et al. (2015) [70]	323 adolescent mothers in Brazil and their children - 163 intervention group - 160 control group - at 4–7 years: - 98 intervention - 109 control	The intervention group received advice on breastfeeding and exclusive breastfeeding and on how to introduce complementary feeding at 6 months.	Breastfeeding and advice on how to start the complementary feeding does not cause a decrease in overweight/obesity prevalence.
Inostroza J et al. (2014) [71]	248 Chilean children born to obese mothers - 172, formula-fed, 86 control group (normal protein) and 86 exp group (low protein) - 76 with breastfeeding (reference group)	- Low protein formula: 1.65 g protein/100 kcal - Control formula 2.7 g protein/100 kcal	Infants fed with low-protein formula have lower weight gain than those fed with a normal formula.
Environmental factors regulating adipose tissue programming			
Iguacel I et al. (2018) [72]	1031 Spanish children	Observational	Rapid infant weight gain, parental overweight/obesity, maternal smoking, and origin/ethnicity predict childhood overweight/obesity and have cumulative effects.
Taylor RW et al. (2017) [76]	206 women at the end of pregnancy—their babies	Recommendations for following an introduction of complementary feeding based on ‘baby-led weaning’.	No differences were shown, although the intervention group showed less anxiety about food.
Early screening for risks associated with high adipose tissue in offspring			
Mitanchez D et al. (2015) [77]	Mini Review		The current definition of GDM does not allow identifying pregestational diabetes from true GDM.
Tam WH et al. (2017) [78]	970 mothers from Hyperglycemia and Adverse Pregnancy Outcome study and their children.	Reevaluations 7 years after delivery.	Maternal hyperglycemia in pregnancy is independently associated with offspring’s risk of abnormal glucose tolerance, obesity, and higher BP at 7 years of age.
McIntyre HD et al. (2015) [79]	Review	To evaluate the different ways of DMG diagnosis	
Corcoran SM et al. (2018) [82]	248 women deemed at risk of GDM before 15 weeks		First trimester measurement of Adiponectin and 1,5-Anhydroglucitol are potential early biomarkers for the later onset of GDM.
Thagaard IN et al. (2017) [83]	2590 pregnant women, categorized into normal weight, moderately obese, or severely obese.		Low adiponectin measured in the first trimester is associated with the development of GDM; higher BMI was associated with lower performance of adiponectin, though this was insignificant. Leptin had an inverse relationship with GDM in severely obese women and did not improve the ability to predict GDM.
Thaware PK et al. (2019) [84]	100 women in early pregnancy from Belfast, UK.	Observational	Ultrasonography-measured visceral adipose tissue in early pregnancy is a potential clinical tool for improving sensitivity of selective screening for gestational diabetes, which, compared with universal oral glucose tolerance testing, is likely to reduce by half the numbers requiring this test.
Alves JG et al. (2020) [85]	627 pregnant women from Brazil.	Prospective cohort	There is an increased risk of GDM in relation to VAD measured in early pregnancy. This association remained so after adjusting for BMI, and VAD was more predictive of GDM than pre-pregnancy BMI.
D’Ambrosi F et al. (2020) [86]	- 238 non-diabetic women - 29 women with 1st trimester GDM - 28 women with 2nd trimester GDM.	Cohorts	Sonographic thickness of maternal visceral adipose tissue was greater in women with GDM than in non-diabetic patients, independently of other known risk factors associated with GDM in the 1st and in the 2nd trimester of pregnancy. Thus, this measurement may be considered of clinical use in 1st trimester screening.
Ma’ayeh M et al. (2020) [94]		Review	Contemporary research into prophylactic and therapeutic interventions for preeclampsia are providing novel and promising modalities.
Kuchenbecker WK et al. (2014) [97]	53 women with obesity and infertility	Prospective cohort	In women with obesity and infertility, measuring IAF by US is in good agreement with the CT scan methodology but the measurement of SAF by US is unreliable.
Pétursdóttir Maack H et al. (2021) [96]	3777 women at around 18 gestational weeks.		Greater subcutaneous adipose tissue thickness measured with second trimester ultrasound is associated with increased risk of developing pre-eclampsia. The measurement may improve prediction models for pre-eclampsia.

**Table 3:** Criteria for the diagnosis of the main obstetric and diabetic associations.

Association	Target Population	Cut-Offs for GMD Diagnosis
FIGO (at any time of gestation) IADPSG (24–28 weeks of gestation)	Universal Risk-based	Fasting glycemia: 92–125 mg/dL (5.1–6.9 mM) Glycemia 1 h after overload ≥ 180 mg/dL (10.0 mM) Glycemia 2 h after overload: 153–199 mg/dL (8.5–11.0 mM)
ACOG NIH (24–28 weeks of gestation)	Risk-based	Step 1: If glycemia ≥ 130 mg/dL (7.8 mM), proceed with Step 2: Fasting glycemia ≥ 95 mg/dL (5.3 mM) Glycemia 1 h after overload ≥ 180 mg/dL (10.0 mM) Glycemia 2 h after overload ≥ 155 mg/dL (8.6 mM) Glycemia 3 h after overload ≥ 140 mg/dL (7.8 mM)
ADA 2020 (24–28 weeks of gestation)	Universal	One step method: 75 g OGTT, fasting postprandial glucose ≥ 92 mg/dL (≥5.1 mmol/L); 1 h: ≥ 180 mg/dL (≥10 mmol/L); 2 h: 153–199 mg/dL (≥8.5 mmol/L). Two step method: 1. First OGTT 50 g of glucose load: fasting postprandial glucose ≥ 95 mg/dL (≥5.3 mmol/L); 1 h: ≥ 180 mg/dL (≥10 mmol/L); 2 h: ≥155 mg/dL/≥8.6 mmlo/L); 3 h: ≥140 mg/dL (≥7.8 mmol/L).
WHO (at any time of gestation)	Universal	One-step method: OGTT (75 g glucose) Two steps method: 1. first OGTT with 50 g of glucose load; >7.8 mmol/L (>1.4 g/L) after 1 h. 2. Second OGTT with 75 g of glucose and evaluation as standard OGTT.

ACOG—American College of Obstetricians and Gynecologists; ADA—American Diabetes Associations; FIGO—International Federation of Gynecology and Obstetrics; IADPSG—International Associations of the Diabetes and Pregnancy Study Groups; NIH—National Institutes of Health; OGTT—oral glucose tolerance test; WHO—World Health Organization. Universal: for general population; Risk-based: screening only for people who meet the criteria for the at-risk population.

## 4. Conclusions

Environmental factors, especially maternal nutrition, have a crucial impact on the fetus and affect adiposity not only at birth but also in the first years of life. Although there are scarce studies performing lasting follow-up, it could be observed how they program the predisposition towards certain non-communicable diseases such as obesity and overweight at later ages such as adolescence and even adulthood. Additionally, there is a strong association between changing dietary patterns and improved adiposity in offspring, especially in overweight women. On the other hand, as regards breastfeeding, there are no differences in adiposity between exclusively breastfed and formula-fed infants, although breastfeeding is still considered the “gold standard”, as it has multiple benefits for the cognitive, immune, and growth development of the newborn, although it does not have a key influence on adiposity.

Some of the factors that are related to a higher risk of intrauterine growth retardation, macrosomia, or being small for gestational age, and which are related to a higher risk of obesity in later stages such as childhood or adolescence are the development of gestational diabetes mellitus or pre-eclampsia, pathologies under study since their prediction in early pregnancy can help to better screen and detect them and therefore begin to treat them from the beginning in order to mitigate their possible effects on the newborn. Therefore, continuing in this line of research will be of great interest in order to reach an agreement that unifies the criteria for the detection of pregnancy-related pathologies and thus be able to reduce the risks associated with them.

Avoiding complications and diseases during pregnancy, such as gestational diabetes mellitus or pre-eclampsia, are some of the simplest strategies for achieving a healthy pregnancy, optimal fetal development, and thus better long-term health of the newborn. Thus, good support during pregnancy, in which mothers are given tools and knowledge about dietary habits and a healthy lifestyle in general, could help reduce the incidence of these complications. It would not only be interesting to provide support during pregnancy but also during breastfeeding and the introduction of complementary feeding, encouraging it in such a way that it is as easy as possible for the mother to carry it out, with nurses and nutritionists who are experts in the field to guide the mother and help her not to give up due to misinformation about how to do it and the benefits it has for the health of her child.

Finally, it should be highlighted the importance of a healthy diet and lifestyle not only during pregnancy and the first months of life but also throughout childhood, especially during the first two years of life as this is a period of great plasticity, preventing the onset of non-communicable diseases such as obesity, diabetes mellitus 2, hypertension, overweight, and any other pathology linked to metabolic syndrome. For all of the reasons mentioned above, to achieve long-term consistent outcomes and prevent the perpetuation of metabolic illnesses, health strategies must be implemented during critical stages of development, such as pregnancy, lactation, and childhood.

## Figures and Tables

**Table 1:** Principal adipokines and their functions.

Adipokine	Biological Function	References
Leptin	Levels during pregnancy are associated with adequate fetal growth. Pro-oxidant role.	Walsh J.M. et al., 2014 [25]
Adiponectin	High levels in lean women, limits placental nutrient transfer and fetal growth compared to obese women. Antidiabetic, anti-inflammatory and cardioprotective.	Sivan E. et al., 2003 [26]
Resistin	It prevents glucose uptake by adipocytes, increasing plasma glucose and leading to insulin resistance. Its levels are increased during pregnancy.	Chen D. et al., 2005 [27]
Angiotensin	Its production is increased under conditions of obesity.	Merrill D. et al., 2002 [28]
IL-6	Elevated levels are associated with higher birth weight. It has a pro-inflammatory role and increases insulin resistance.	Aye I. L. et al., 2015 [29]
IL-10	Has anti-inflammatory effects.	Houra M. et al., 2021 [30]
TNF-α	It is associated with higher birth weight in newborns. It is a pro-inflammatory cytokine.	Hunt J.S. et al., 1996 [31]
MCP-1	Increased levels in mothers with obesity. Pro-inflammatory effects.	Rull A. et al., 2010 [32]
Grelin	Increases appetite by modulating orexigenic peptides in the hypothalamus. Coordinates energy balance and weight regulation.	Kierson J. A. et al., 2006 [33]
Apelin	Anti-diabetic and anti-obesogenic properties.	Boucher J. et al., 2005 [34]
ASP	Inhibits hormone-sensitive lipase and leads to increased insulin release from beta cells.	Cianflone K. et al., 2003 [35]
SAA	Directly measures inflammation associated with obesity.	Ostensen M. G. et al., 1985 [36]

IL-6—interleukine-6; IL-10—interleukine-10; TNF-α—tumor necrosis factor-α; MCP-1—monocyte chemoattractant protein 1; ASP—acylation stimulating protein; SAA—salivary alpha-amylase.

## Data Availability

Not applicable.
